# Supplementary material for: WordCommentsAnalyzer: A windows software tool for qualitative research
Source: F1000Res. 2018 Sep 4;7:536. Originally published 2018 May 3. [Version 2] doi: 10.12688/f1000research.14819.2 (PMC6137411; doi:10.12688/f1000research.14819.2)
Supplement: Supplementary file 3 [file f1000research-7-17614-s0002.tgz › b2a79496-4ada-47c4-9682-a99ce867836d.zip!/Eight Iranian Health Journals/Journal of Knowledge and Health_27.docx]

© Journal of Knowledge and Health. This work is licensed under CC BY 4.0 (https://creativecommons.org/licenses/by/4.0/).

Volume & Issue: دوره 6، شماره 3: 1390

Title: اعتبار عاملی و ویژگی‌های روان‌سنجی پرسش‌نامه فرسودگی شغلی ماسلاچ (نسخه فارسی)

http://knh.shmu.ac.ir/index.php/site/article/view/99

مقدمه: پرسش‌نامه فرسودگی شغلی ماسلاچ (Maslach Burnout Inventory)، متداول‌ترین ابزار سنجش فرسودگی شغلی در میان افراد مختلف با سوابق شغلی و حرفه‌ای متفاوت است. نسخه آموزشگران این پرسش‌نامه به‌طور خاص برای بررسی فرسودگی شغلی در میان کسانی که در امر آموزش و یاددهی دخیل‌اند طراحی شده است. مواد و روش‌ها: پرسش‌نامة فرسودگی ماسلاچ به زبان‌های مختلفی ترجمه شده است و ساختار عاملی آن در مطالعات متفاوتی مورد بررسی قرار گرفته است. در این مطالعه، ساختار عاملی نسخه فارسی MBI با استفاده از روش‌های تحلیل عاملی اکتشافی و تحلیل عاملی تأييدی در میان 570 معلم زبان انگلیسی مورد بررسی قرار گرفته است. نتایج: یافته‌های اين پژوهش در مورد نسخۀ فارسی پرسش‌نامۀ فرسودگی شغلی، نشان‌دهندة صحت مدل سه عاملی که در اصل توسط ماسلاچ و جکسون ارائه شده، می‌باشد. نتیجه‌گیری: با توجه به نتایج به‌دست آمده، پرسش‌نامه فارسی فرسودگی شغلی قابلیت استفاده در تحقیقات فرسودگی شغلی در ایرانیان فارسی زبان را دارد.

Title: اثربخشي درمان شناختي- رفتاري مديريت استرس بر سلامت عمومي بيماران مبتلا به آسم

http://knh.shmu.ac.ir/index.php/site/article/view/101

مقدمه: درمان‌هاي روانشناختي مي‌توانند سهم بسزايي در بهبود سلامت عمومي بيماران مبتلا به آسم داشته‌ باشند. هدف از انجام اين پژوهش، بررسي اثر بخشي درمان شناختي- رفتاري مديريت استرس، به شيوه‌‌‌‌‌‌ي گروهي بر سلامت عمومي بيماران مبتلا به آسم است. مواد و روش‌ها: در اين مطالعه مداخله‌اي، 24 بيمار زن مبتلا به آسم انتخاب و به‌صورت تصادفي به دو گروه مداخله و شاهد تخصيص داده شدند. براي جمع‌آوري داده‌ها از پرسش‌نامه "سلامت عمومي- 28" استفاده شد كه در پيش‌آزمون، پس‌آزمون و پيگيري، توسط افراد تكميل گرديد. درمان شناختي- رفتاري مديريت استرس، طي 8 جلسه بر روي گروه مداخله انجام گرفت و گروه شاهد ضمن دريافت درمان‌هاي متداول پزشکي، جهت درمان‌ روانشناختي در ليست انتظار قرار گرفت. داده‌ها با استفاده از آزمون تحليل كواريانس مورد تجزيه‌وتحليل آماري قرار گرفتند. نتایج: يافته‌هاي به‌دست‌آمده نشان داد که ميانگين نمرات سلامت روان در گروه مداخله (پيش‌آزمون= 8/37، پس‌آزمون= 3/30 و پيگيري= 7/35) کاهش معناداري در مراحل پس‌آزمون و پيگيري نسبت‌به گروه شاهد (پيش‌آزمون= 2/38 پس‌آزمون= 9/39 و پيگيري= 7/41) داشته است (0001/0=P). نتیجه‌گیری: نتايج مطالعه حاضر حاکي از اثربخشي درمان شناختي- رفتاري مديريت استرس بر بهبود سلامت عمومي بيماران زن مبتلا به آسم است. با توجه به کاهش سلامت روان در بیماران مبتلا به آسم و مؤثربودن مداخلة، رواني لزوم توجه جدي به درمان‌هاي روان‌شناختي در اين گروه بيماران ضروری مي‌نمايد.

Title: عوامل مؤثر بر اجرای فرآیند پرستاری توسط پرستاران: یک مطالعه کیفی

http://knh.shmu.ac.ir/index.php/site/article/view/102

مقدمه: فرآیند پرستاری، روشی سیستماتیک و پویا است که کاربرد آن به ارتقاء کیفی مراقبت از بیمار کمک می‌کند. مروری بر متون مختلف و مرتبط، نشان می‌دهد اجرای فرآیند پرستاری نه تنها در ایران بلکه در بسیاری از کشورهای جهان با چالش‌های بسیاری روبه‌رو است. به کارگیری فرآیند پرستاری مستلزم درک عوامل مؤثر بر اجرای آن از دیدگاه پرستاران و چگونگی تأثیر آن است. هدف از این مطالعه کشف عوامل مؤثر بر اجرای فرآیند پرستاری توسط پرستاران است. مواد و روش‌ها: در اين مطالعه كيفي، از رویکرد نظریه پایه برای کشف دیدگاه پرستاران درمورد اجرای فرآیند پرستاری استفاده شد. به این منظور 18 پرستار بالینی، 12 مربی پرستاری و 6 مدیر پرستاری در مصاحبه‌های نیمه ساختار یافته شرکت نمودند و داده‌ها به‌طور هم‌زمان از طریق مقایسه مداوم تحلیل شد. نتایج: عامل فردی- ‌مدیریتی به‌عنوان متغیر اصلی این پژوهش نمایان‌‌ شد. این عامل بدین معناست که باید مجموعه‌ای از عوامل فردی و مدیریتی مهیا گردند تا به پرستاران در به‌کارگیری فرآیند پرستاری به‌منظور ارتقاء کیفیت مراقبت‌ها، کمک کنند. این‌ متغیر شامل دو کد محوری‌ فردی و مدیریتی است. در سطح فردی سه طبقه (آگاهي، نگرش و مهارت) و در سطح مدیریتی پنج طبقه (نظارت، نيروي انساني كافي، تشويق و تنبيه، ابزار و شرايط مناسب و مشاركت گروهي) در نظر گرفته شد. نتیجه‌گیری: عوامل مؤثر بر اجرای فرآیند پرستاری توسط پرستاران، متنوع و پیچیده بوده و مربوط به عوامل فردی و مدیریتی است. بنابراین، مدیران پرستاری و مسئولین آموزش پرستاری باید تلاش کنند تا موانع‌ موجود را رفع نموده و تسهیلاتی براي ارتقای‌ اجرای فرآیند پرستاری و افزایش ارائه خدمات با کیفیت مبتنی بر فرآیند پرستاری را فراهم نمایند.

Title: بررسی دیدگاه‌ها و عقاید زنان درمورد موانع استفاده از IUD: یک مطالعه كيفي

http://knh.shmu.ac.ir/index.php/site/article/view/104

مقدمه: استفاده از IUD يكي از رايج‌ترين روش‌هاي ضد بارداری در سراسر دنیا است. طبق آمار ارائه شده در نظام نوین ادغام یافته مدیریت و ارزشیابی تحقیقات ملی ایران IMES (The Integrated Monitoring Evaluation System)، استفاده از IUD در ایران 9/11% بوده است، ولی این رقم در خمین 4% می‌باشد که در مقایسه با آمار کشوری بسیار کم است. هدف ازانجام این مطالعه، تبیین دیدگاه و عقاید زنان شهرستان خمین درمورد موانع استفاده از IUD به‌عنوان روش پیشگیری از بارداری می‌باشد. مواد و روش‌ها: پژوهش حاضر یک مطالعه کیفی به‌صورت بحث گروه‌های متمرکز است. جمع‌آوری اطلاعات از زنان سنین 15-49 ساله، داراي همسر‌، واجد شرایط استفاده از روش‌های پیشگیری و ساکن روستاهای خمین انجام شد. در جلسات یک نفر گرداننده بحث و یک نفر ثبت‌کننده صحبت‌های شرکت‌کنندگان حضور داشت. آنالیز داده‌ها به‌روش تحلیل محتوا انجام شد. نتایج: اکثریت شرکت‌کنندگان تمایل به استفاده از IUD نداشتند. مهم‌ترین دلایل عدم‌تمایل آنان عوارضIUD و باورهای غلط بود. دلایل دیگر شامل احساسات (ترس و نگرانی از پروسه صحیح IUD گذاری) و عدم‌اطلاع از ظاهر و مزایا و معایب آن، تأثير نظرات دیگران (پرسنل بهداشتی و...) و موانعی از قبیل عدم‌رضایت همسر بودند. نتیجه‌گیری: با توجه به نقش تأثيرگذار پرسنل بهداشتی و همسر و هم‌چنین تأثير تبحر ماما و پزشک در انتخاب نوع روش پیشگیری از بارداری و ازطرفی اطلاعات ضعیف واجدین شرایط تنطیم خانواده درمورد IUD، ضرورت هر چه بیش‌تر آموزش IUD گذاری صحیح و ارتقای مهارت انجام مشاوره تنظیم خانواده در افراد فوق‌الذکر و مشارکت دادن مردان در امر تنظیم خانواده، کاملاً محسوس است.

Title: شیوع اختلالات عملکردی روده در استان تهران: مطالعه مبتنی بر جمعیت

http://knh.shmu.ac.ir/index.php/site/article/view/105

مقدمه: برآورد شیوع اختلالات عملکردی روده و زیرگروه‌های آن به‌طور چشمگیری با تشخیص بیماری ارتباط مستقیم دارد. این بررسی با هدف برآورد شیوع و عوامل خطر اختلالات عملکردی روده در بین جامعه ایرانی طرح‌ریزی و اجرا شده است. مواد و روش‌ها: این مطالعه از نوع مطالعات مقطعی می‌باشد که بر پایه پرسش‌نامه اعتبار یافته بر‌اساس معیار RomeIII، از اردیبهشت 1385 تا آذر 1386، جهت برآورد شیوع علائم و اختلالات گوارشی بر روی 18180 نفر ساکن شهر تهران که به‌صورت تصادفی انتخاب شده بودند، انجام شد. نتایج: در مجموع بر‌اساس معیار RomeIII‌، 1/1% افراد مبتلا به سندرم ‌روده تحريك‌پذير، 5/1% افراد مبتلا به نفخ عملکردی، 4/2% افراد مبتلا به یبوست عملکردی، 5/5% افراد مبتلا به اختلالات نامشخص عملکردی روده، 4/0 افراد مبتلا به اسهال عملکردی و 9/10% از افراد مورد مطالعه حداقل دارای یکی از اختلالات عملکردی روده بودند. نکته جالب توجه این است که 8/83% از افرادی که سوء هاضمه عملکردی، 6/78% از افرادی که اسهال، 2/77% از افرادی که نفخ، 5/74% از افرادی که يبوست و 9/72% از افرادی که درد شکمی را به‌صورت خود اظهاری گزارش کرده بودند، دارای اختلالات عملکردی روده بودند. نتیجه‌گیری: این مطالعه نشان‌دهنده شیوع پايين اختلالات عملکردی روده در بین جمعیت شهری استان تهران می‌باشد. نواقص موجود در معیار RomeIII و مشکلات مربوط به تفسیر و جمع‌آوری اطلاعات، خود می‌توانند عللی در تخمین کم شیوع اختلالات عملکردی روده باشند. علاوه بر این قابلیت اعتماد به یادآوری علائم در شش ماه گذشته بر‌اساس RomeIII برای جمعیت مورد مطالعه، سؤال بر‌انگیز است.

Title: تعيين فراواني عفونت هم‌زمان هپاتيت‌های ویروسی ‌B و C با HIV و عوامل مؤثر بر آن

http://knh.shmu.ac.ir/index.php/site/article/view/106

مقدمه: یکی از مشكلات همراه در بیماران مبتلا به HIV، ابتلا به انواع هپاتيت C ,B در مي‌باشد. با توجه به تأثیرات سوء عفونت توأم این سه ویروس در بدن و باتوجه به این که تاکنون مطالعات اندکی درمورد فراواني انواع هپاتيت C ,B در مبتلايان به HIV در كشور ما انجام شده است، ما در این مطالعه به بررسي فراواني عفونت هپاتیت B (HBV) و C‌ (HCV) در مبتلايان به HIV و عوامل مرتبط با آن پرداختیم. مواد و روش‌ها: با انجام مطالعة مقطعی، فراوانی عفونت توأم HCV /HIV/HBV و عوامل مرتبط با آن در 365 بیمار مورد مطالعه قرار گرفت. بیماران در مركز مشاوره بيماري‌هاي رفتاري بيمارستان امام خمینی (ره)، طي سال‌هاي 88- 83، جهت تعيين فراواني عفونت توأم هپاتيت C ,B با HIV مورد بررسی قرار گرفتند. مشخصات دموگرافیک همراه با چگونگي ابتلا، سابقه واکسیناسیون هپاتیت B، مرحله بيماري و اندازه‌گیری سطح HBs-Ab، از پرونده بیماران استخراج گردید. نتایج: در مجموع 3/26% افراد مطالعه، عفونت توأم HCV/HIV/HBV را داشتند. بیشترین رده سنی افراد با عفونت توأم، 188 نفر (5/51%)، بین 21 تا40 سال بودند. 9/34% افراد با عفونت توأم از طریق اعتیاد تزریقی مبتلا شده بودند. 1/30% از افراد با عفونت توأم در مرحله‌ی نهفتگی بیماری بودند. 3/22% از بیماران با عفونت توأم سابقه‌ی واکسیناسیون هپاتیت B را داشتند و 7/30% از افراد با عفونت توأم، HBs-Ab مثبت بودند. نتیجه‌گیری: بر‍‌اساس نتایج به‌دست آمده توصیه می‌شود که بیماران HIV مثبت، واکسن هپاتیت B را دریافت نمایند. هم‌چنین بر استفاده از رژیم ضدرتروویروسی خیلی فعال (HAART) در موارد عفونت توأم برای کاهش اثرات مضر ویروس‌های هپاتیت بر کبد تأکید می‌گردد.

Title: بررسي ميزان آمادگي بيمارستان‌هاي استان سمنان در مقابله با بلايا

http://knh.shmu.ac.ir/index.php/site/article/view/108

مقدمه: بيمارستان‌ها در زمان بروز بلايا از اصلي‌ترين واحدهاي ارائه خدمات بهداشتي درماني بوده كه آمادگي و ارائه خدمات به‌موقع آن‌ها، در كاهش صدمات و مرگ‌ومير نقش حياتي دارد. اين مطالعه به‌منظور تعيين ميزان آمادگي بيمارستان‌هاي استان سمنان در مقابله با بلايا انجام‌شده‌است. مواد و روش‌ها: در اين مطالعه كليه مديران ارشد بيمارستان‌هاي استان سمنان در سال 1389 به‌روش سرشماري انتخاب و پرسش‌نامه‌اي حاوي 40 سؤال درزمينه آگاهي مدیریت از وضعیت بيمارستان و چک‌لیستی شامل 133 سؤال اختصاصی به‌صورت مصاحبه و مشاهده مستندات تکمیل شد. جهت مقايسه ميانگين نمرات در بين گروه‌ها، از آزمون آناليز واريانس استفاده شد. نتايج: از 10 بیمارستان مورد مطالعه، 50% آموزشی- درمانی و مابقی فقط درمانی بودند. ميانگين درصد آمادگي بيمارستان‌ها در حيطه‌هاي پشتیبانی خدمات حیاتی 80%، مديريت حوادث غیرمترقبه در بیمارستان 65%، برنامه‌ریزی اقدامات بهداشت محیط بیمارستان برای مقابله با بلایا 2/56%، ايمني تجهيزات و مواد خطرناك 2/64%، كاهش خطرات ساختماني 8/43%، تخلیه بیمارستان و درمان صحرایی 5/49% و برنامه‌ریزی آموزشی بیمارستان برای مقابله با بلایا 2/42% بود. بین امتیاز آگاهی مدیر و آمادگی بیمارستان رابطه معناداری مشاهده گردید (001/0=P). در مجموع بيمارستان‌هاي استان با ميانگين 7/15±4/58 درصد، از سطح آمادگي متوسط در مقابله با بلايا برخوردار بودند. نتيجه‌گيري: با عنايت به نتايج و اعلام نياز تمامي مديران به آموزش درزمينه بلايا، بازآموزي مديران در كليه زمینه‌ها ازجمله بهداشت محیط، كاهش خطرات ساختماني، تخلیه بیمارستان و درمان صحرایی و برنامه‌ریزی آموزشی بیمارستان برای مقابله با بلایا ضروري است.

Title: بررسي نشانه‌هاي درد در نوزادان

http://knh.shmu.ac.ir/index.php/site/article/view/110

مقدمه: درد يك تجربه مخرب و صدمه‌زا است و در خاطر نوزاد دچار درد، باقي مي‌ماند. طيف بررسي‌هاي انجام شده طبق اين واكنش‌ها نسبت‌به محركات دردناك، بسيار گسترده بوده و لذا شناخت و بررسي آن‌ها مي‌تواند علاوه بر كمك به نوزادان، تغييراتي را در پروسيجرهاي دردناك پزشكي و پرستاري در قبال نوزادان ايجاد نمايد. از اين روي اين تحقيق با هدف تعيين نشانه‌هاي رفتاري و فيزيولوژيكي درد در نوزادان بستري در بخش نوزادان بيمارستان امام حسين (ع) در سال 87 انجام شد. مواد و روش‌ها: اين پژوهش يك مطالعه توصيفي بوده كه با بررسي كليه نوزادان واجد شرايط‌ شركت در مطالعه كه در بخش نوزادان بيمارستان امام حسين (ع) در شش ماهه اول سال 87 بستري بودند، صورت گرفت. جهت گردآوري داده‌ها از چك ليست استاندارد كه بر اساس تئوري سيناكيتو تكاملي آلس تهيه شده و ابزار استاندارد بررسي درد نوزاد ، ساخته لورنس، استفاده شد. نتایج: از 141 نوزاد مورد بررسی، 63 نفر (7/44%) مؤنث و 78 نفر (3/55%) مذکر بودند. میانگین سنی نوزادان 5/6±2/7 روز (با دامنه 1- 17 روز) و میانگین وزن نوزادان 6/258±2/3015 گرم (با دامنه2250- 3950 گرم) بود. در‌خصوص علت بستری، نوزادان ایکتر با 78 مورد (3/55%) بالاترین علت بستری را شامل شدند. تغذیه از شیر مادر در 6/71% نوزادان صورت گرفته و 78% نوزادان حاصل زایمان سزارین بودند. در بین پروسیجرهای پزشکی، خونگیری در همه موارد انجام شده است. در‌خصوص نشانه‌های رفتاری، تغییر حالت چهره با 8/96% بالاترین میزان را داشته و بعد تغییر الگوی تنفسی با 8/94% قرار گرفته است. میانگین امتیاز کسب شده نوزادی 85/1±21/6 امتیاز (با دامنه 2-7) بود. تغییرات ضربان قلب و تنفس با پروسیجرهای انجام شده، رابطه معناداری نشان دادند. نتیجه‌گیری: با توجه به نتایج حاصل می‌توان به نشانه‌های رفتاری و فیزیولوژیکی نوزادان برای بررسی درد اعتماد نمود و با تهیه فرم‌های ‌مناسبت‌تر بررسی رفتاری درد با نشانه‌های فیزیولوژیک، کارایی این ابزارها را ارتقاء داد.
